# Clinical Aspects of Infectious Diseases

**DOI:** 10.3390/jcm13164853

**Published:** 2024-08-16

**Authors:** Yasir Waheed

**Affiliations:** 1 Gilbert and Rose-Marie Chagoury School of Medicine, Lebanese American University, Byblos 1401, Lebanon; yasir_waheed_199@hotmail.com; 2 MEU Research Unit, Middle East University, Amman 11831, Jordan

## 1. Introduction

Infectious diseases are illnesses caused by harmful pathogens, including viruses, bacteria, fungi, and parasites. Humans face ever-evolving challenges with infectious diseases.

The coronavirus pandemic has disrupted healthcare and financial systems, with over 704 million cases and over 7 million deaths [1].

Currently, more than 300 million people suffer from hepatitis B and C infections worldwide. Every day, around 6000 new people are infected with viral hepatitis. Medicines are available for the treatment of viral hepatitis at affordable rates, but many countries do not take advantage of these treatments because of barriers to accessing policies, and programmatic issues. The majority of nations are not on track to meet the World Health Organization’s 2030 target of eliminating viral hepatitis [2].

In the last two decades, significant progress has been made in the fight against HIV/AIDS. Approximately 36.8 million people suffer from HIV infection worldwide. Sub-Saharan Africa has the highest burden of HIV, and the region has made significant progress in its fight against HIV [3].

The World Health Organization (WHO) developed a strategy to end TB, called End TB, with 2020 interim targets that included a 20% reduction in TB incidence and a 35% reduction in TB mortality. However, only 15 out of 204 countries attained a 20% reduction in the incidence of TB, and only 17 countries achieved a 35% reduction in TB mortality from 2015 to 2020. It is time for countries to revisit their End TB targets to achieve TB control by 2035 [4].

In addition, according to the Center for Disease Control (CDC), nearly half of the world population is at risk of contracting malaria. Since 2000, global malaria deaths have been reduced to half, thereby saving 11.7 million lives worldwide [5].

## 2. Overview of Published Articles

Sekowska et al. studied Klebsiella pneumoniae infection in 1921 children experiencing cancer therapy and 1697 children undergoing hematopoietic cell transplantation in Poland. A total of 527 children developed K. pneumoniae infection, with prevalence rates of 4.86% and 4.95%, in children undergoing cancer therapy and hematopoietic cell transplantation, respectively. The prevalence of multidrug-resistant K pneumoniae strains was high, and 3.22% of children died from K. pneumoniae infection (Contribution 1). Moreover, 55.2% of children also developed urinary tract infections.

Bloodstream infections (BSIs) that occur in intensive care units (ICUs) worsen the condition of patients who are already very sick, increasing their risk of morbidity and death. Kokkoris et al. conducted a study to analyze the role of C-reactive proteins (CRPs), prolactin (PCT), and the neutrophil–lymphocyte ratio (NLR) in differentiating between ICU-acquired candidemia and bacteremia. The study was conducted on 63 patients, 31 of whom had candidemia and 32 with bacteremia. The CRP, NLR, and PT values were significantly lower in patients with candidemia than in those with bacteremia. The study concluded that routinely available laboratory tests, including CRP, PT, and NLR, can be used for the early detection of candidemia in ICU patients (Contribution 2).

Ear, nose, and throat (ENT) symptoms are experienced by individuals who are HIV-positive during their infection. The most common ENT manifestations are sinusitis and otitis, which are more frequent in patients with a CD4+ T-cell count below 200 cells/µL. Plesca et al. analyzed the hospitalization of patients with HIV due to acute otitis (AO) and acute sinusitis (AS). A total of 179 cases were analyzed in the study, out of which, 149 were hospitalized due to AS and 41 were hospitalized due to AO. A total of 57.5% of the patients with HIV enrolled in the study were classified as stage C3. Most of the hospitalizations occurred due to infection by four bacterial pathogens, including *Staphylococcus aureus*, *Haemophilus influenza*, *Pseudomonas aeruginosa*, and *Streptococcus pneumoniae* (Contribution 3).

Myalgic encephalomyelitis/chronic fatigue syndrome (ME/CFS) is a complex neurological disorder with an etiology that is not fully understood. Confirmed or probable viral infections are found in many patients before the onset of symptoms. Prior to COVID-19, many ME/CFS patients showed Epstein–Barr virus (EBV)-associated mono nucleases as a common cause. Christa et al. conducted a pilot study to assess ME/CFS in 174 patients, including 130 adults and 43 pediatric patients. Viral triggers were identified in 92% of patients, and half of the patients showed COVID-19 infection as the triggering agent. These patients showed severe functional and social impairment and decreased health-related quality of life (Contribution 4).

In emergency departments, urinary tract infections (UTIs) are the leading type of bacterial infections. Culture-based diagnostic testing takes 24–48 h, and in some cases, a fast and precise diagnosis is required. Hertz et al. conducted a study to analyze the precision of C-reactive protein (CRP), soluble urokinase plasminogen activator receptors (suPARs), and procalcitonin (PCT) in diagnosing UTIs. The study concluded that suPAR had little diagnostic value in patients with UTIs, whereas PCT and CRP could not be used for the diagnosis of UTIs (Contribution 5).

One of the top 10 major causes of death worldwide is tuberculosis (TB). Approximately 80% of TB cases affect the lungs, and the remaining 20% affects other parts of body, such as lymph nodes, pleura, skin, joints, bones, the nervous system, and the genitourinary track. Neurotuberculosis is the least common, but most devastating, form of TB. Corona-Nakamura et al. conducted a study on 103 intraspinal and 82 intracranial neuro TB patients. In-hospital mortality was reported in 29.6% of intracranial and 3% of intraspinal neuro TB patients. The risk factors of intraspinal neuro TB mortality were diabetes mellitus, an age of more than 40 years, and diagnostic delays. The risk factors for intracranial neuro TB were HIV infection, stroke, hydrocephalus, lymphopenia, etc. (Contribution 6).

Community-acquired pneumonia (CAP) is a leading cause of mortality in industrialized countries. Candel et al. reviewed important issued linked with CAP diagnostics, treatment, and vaccination. Real-time PCR-based diagnostic panels are available for the detection of a wider range of bacterial and viral pathogens, as observed for CAP. For a duration of 5–7 days, combination therapy consisting of a beta-lactam and a macrolide is highly recommended. An excessive oxygen supply is useful for the management of hypoxia. Steroid treatment must be individualized because it has a positive impact on distress and shock situations and may have a negative impact on immunological control of the infection. Resistant microorganisms are the most common cause of treatment failure in community-acquired pneumonia. Both immunocompromised and immunocompetent patients demonstrated improvements in the incidence of pneumonia and death after a preventive vaccination (Contribution 7).

Hospital-acquired pneumonia (HAP) is an infection that develops 48 h after hospital admission. An elevated risk of death is linked to HAP and should be treated by a team of different specialties, including intensive care physicians, pulmonary disease physicians, infectious diseases physicians, anesthesiologists, microbiologists, and other healthcare professionals. HAP is primarily caused by four pathogens, namely *Klebsiella pneumoniae*, *Escherichia coli*, *Pseudomonas aeruginosa*, and *Staphylococcus aureus* (Contribution 8).

Vulvovaginal candidiasis (VVC) is a fungal infection of the vagina and vulva. *Candida albicans* and non-albicans species, such as *C. tropicalis*, *C. parapsilosis*, *C. glabrata*, and *C. krusei*, are the main causative agents. In the United States, VVC is a serious issue that impacts 70–75% of women at some point in their lives. The treatment of VVC depends upon the form of the disease. VVC treatment may include oteseconazole and ibrexafungerp. Tropical or oral azoles are recommended for uncomplicated mycosis; probiotics, TOL-463, and chlorhexidine have also shown satisfactory results. Fluconazole can be used to treat recurrent VVC (Contribution 9).

Globally, colorectal cancer (CRC) ranks third in terms of cancer-related mortality. A diet deficient in fruits and vegetables, obesity, smoking, and physical inactivity are risk factors for CRC. Given the poor prognosis of CRC, new prognostic and diagnostic indicators must be developed to detect CRC. Numerous cytokines are generated at different stages of colorectal cancer (CRC), and they can be utilized as biomarkers to detect CRC. Pro-tumorigenic host response associated with genes and proteins, such as TNF, TGF-B, VEGF, IL-4, IL-6, IL-8, IL-11, IL-17a, IL-22, IL-23, and IL-33, can be further explored to analyze their role in CRC detection (Contribution 10).

A zoonotic disease called leptospirosis is caused by direct or indirect contact with animal urine, especially that of rats. Approximately 1.03 million cases of Leptospirosis are reported annually, with 58,900 deaths. Animals emit disease-causing pathogenic Leptospira in their urine, which unintentionally hosts and may become life-threatening reservoirs in humans. Most infections are either asymptomatic or mild. Symptomatic infections usually begin with fever, muscle aches, chills, flu-like symptoms, and headache. Leptospirosis diagnosis is challenging, particularly in resource-limited settings. A systematic review was conducted by Petakh et al. to analyze the role of corticosteroids in the treatment of Leptospirosis. Five studies were analyzed in the review. Four studies indicated a beneficial role of corticosteroid and one randomized study showed no significant benefit of corticosteroid in the treatment of Leptospirosis (Contribution 11).

One of the main hospital-acquired infections that causes high morbidity and mortality rates, extended hospital stays, and increased healthcare costs is methicillin-resistant Staphylococcus aureus (MRSA). Chlorhexidine (CHG), which is applied by using wipes or baths, and mupirocin, which is applied via the nose, are the usual methods for MRSA decontamination. A study by Nahra et al. found that hospital-acquired MRSA and CLABSIs in critically ill patients were significantly reduced when 10% povidone iodine (PI) was applied to the nares in addition to CHG bathing (Contribution 12).

Human herpesvirus 7 (HHV-7) typically causes mild and self-limiting infection in children. Ninety percent of children older than five and sixty-five percent of toddlers less than two are seropositive for HHV-7. The most common symptom of the infection is a 3-day-long fever; there is no evidence of congenital HHVV-7 infection. Few cases have been documented where HHV-7 has crossed the blood–brain barrier and affected the central nervous system (CNS). An 11-month-old boy infected with HHV-7 was reported by Moppert et al. Head magnetic resonance imaging (MRI) revealed many diffuse foci of demyelination in the hippocampus, parietal, temporal, and occipital lobes. Treatment with ganciclovir showed significant reduction in demyelination foci. A post-therapy follow-up revealed that the child’s psychomotor development was delayed (Contribution 13).

Cystic echinococcosis causes cyst formation mainly in the liver and lungs and less frequently in the bones, kidneys, spleen, eyes, central nervous system, and muscles. Because the cysts grow slowly, symptoms do not appear for years. Eventually, they may cause nausea, vomiting, or coughing in addition to pain in the upper abdomen or chest. According to Popa et al., a patient with multi-visceral cystic echinococcosis had a difficult clinical course that necessitated substantial medical and surgical care. The majority of post-surgery relapses happen within the first three to six years, which is why the study came to the conclusion that patients with CE require long-term follow-up (Contribution 14).

## 3. Conclusions

Infectious diseases caused by viruses, bacteria, fungi, and parasites continuously create serious problems for humans. There is a need to use the latest diagnostic methods to detect infectious diseases. Timely diagnosis will help initiate appropriate treatment. In complicated infectious diseases, teams from diverse specialties, such as intensive care, pulmonary disease, infectious diseases, anesthesiologists, microbiologists, and other healthcare professionals may be used to save lives. There is also a need to increase financing for the control of major infectious diseases.

## List of Contributions

1. Sękowska, Alicja, Czyżewski, Krzysztof, Jaremek, Kamila, Zalas-Więcek, Patrycja, Zając-Spychała, Olga, Wachowiak, Jacek, Szmydki-Baran, Anna, Hutnik, Łukasz, Gietka, Agnieszka, Gryniewicz-Kwiatkowska, Olga, and et al. 2024. Infections with *Klebsiella pneumoniae* in Children Undergoing Anticancer Therapy or Hematopoietic Cell Transplantation: A Multicenter Nationwide Study. *Journal of Clinical Medicine* 13: 4078. https://doi.org/10.3390/jcm13144078.
2. Kokkoris, Stelios, Angelopoulos, Epameinondas, Gkoufa, Aikaterini, Christodouli, Foteini, Ntaidou, Theodora, Theodorou, Evangelia, Dimopoulou, Georgia, Vasileiadis, Ioannis, Kremmydas, Panagiotis, and Christina Routsi. 2024. The Diagnostic Accuracy of Procalcitonin and Its Combination with Other Biomarkers for Candidemia in Critically Ill Patients. *Journal of Clinical Medicine* 13: 3557. https://doi.org/10.3390/jcm13123557.
3. Pleșca, Vlad Ștefan, Miron, Victor Daniel, Marinescu, Adrian Gabriel, Drăgănescu, Anca Cristina, Pleșca, Anca Doina, Săndulescu, Oana, Voiosu, Cătălina, Hainăroșie, Răzvan, and Anca Streinu-Cercel. 2024. Hospitalizations for Acute Otitis and Sinusitis in Patients Living with HIV: A Retrospective Analysis of a Tertiary Center in Romania. *Journal of Clinical Medicine* 13: 3346. https://doi.org/10.3390/jcm13113346.
4. Hieber, Hannah, Pricoco, Rafael, Gerrer, Katrin, Heindrich, Cornelia, Wiehler, Katharina, Mihatsch, Lorenz L., Haegele, Matthias, Schindler, Daniela, Donath, Quirin, Christa, Catharina, and et al. 2024. The German Multicenter Registry for ME/CFS (MECFS-R). *Journal of Clinical Medicine* 13: 3168. https://doi.org/10.3390/jcm13113168.
5. Hertz, Mathias Amdi, Johansen, Isik Somuncu, Rosenvinge, Flemming S., Brasen, Claus Lohman, Andersen, Eline Sandvig, Heltborg, Anne, Skovsted, Thor Aage, Petersen, Eva Rabing Brix, Cartuliares, Mariana Bichuette, Nielsen, Stig Lønberg, and et al. 2024. The Diagnostic Accuracy of Procalcitonin, Soluble Urokinase-Type Plasminogen Activator Receptors, and C-Reactive Protein in Diagnosing Urinary Tract Infections in the Emergency Department—A Diagnostic Accuracy Study. *Journal of Clinical Medicine* 13: 1776. https://doi.org/10.3390/jcm13061776.
6. Corona-Nakamura, Ana Luisa, Arias-Merino, Martha Judith, Miranda-Novales, María Guadalupe, Nava-Jiménez, David, Delgado-Vázquez, Juan Antonio, Bustos-Mora, Rafael, Cisneros-Aréchiga, Aldo Guadalupe, Aguayo-Villaseñor, José Francisco, Hernández-Preciado, Martha Rocio, and Mario Alberto Mireles-Ramírez. 2023. Intraspinal and Intracranial Neurotuberculosis, Clinical and Imaging Characteristics and Outcomes in Hospitalized Patients: A Cohort Study (2000–2022). *Journal of Clinical Medicine* 12: 4533. https://doi.org/10.3390/jcm12134533.
7. Candel, Francisco Javier, Salavert, Miguel, Basaras, Miren, Borges, Marcio, Cantón, Rafael, Cercenado, Emilia, Cilloniz, Catian, Estella, Ángel, García-Lechuz, Juan M., Montero, José Garnacho, and et al. 2023. Ten Issues for Updating in Community-Acquired Pneumonia: An Expert Review. *Journal of Clinical Medicine* 12: 6864. https://doi.org/10.3390/jcm12216864.
8. Candel, Francisco Javier, Salavert, Miguel, Estella, Angel, Ferrer, Miquel, Ferrer, Ricard, Gamazo, Julio Javier, García-Vidal, Carolina, del Castillo, Juan González, González-Ramallo, Víctor José, Gordo, Federico, and et al. 2023. Ten Issues to Update in Nosocomial or Hospital-Acquired Pneumonia: An Expert Review. *Journal of Clinical Medicine* 12: 6526. https://doi.org/10.3390/jcm12206526.
9. Satora, Małgorzata, Grunwald, Arkadiusz, Zaremba, Bartłomiej, Frankowska, Karolina, Żak, Klaudia, Tarkowski, Rafał, and Krzysztof Kułak. 2023. Treatment of Vulvovaginal Candidiasis—An Overview of Guidelines and the Latest Treatment Methods. *Journal of Clinical Medicine* 12: 5376. https://doi.org/10.3390/jcm12165376.
10. Maryam, Sajida, Krukiewicz, Katarzyna, Haq, Ihtisham Ul, Khan, Awal Ayaz, Yahya, Galal, and Simona Cavalu. 2023. Interleukins (Cytokines) as Biomarkers in Colorectal Cancer: Progression, Detection, and Monitoring. *Journal of Clinical Medicine* 12: 3127. https://doi.org/10.3390/jcm12093127.
11. Petakh, Pavlo, Oksenych, Valentyn, and Oleksandr Kamyshnyi. 2024. Corticosteroid Treatment for Leptospirosis: A Systematic Review and Meta-Analysis. *Journal of Clinical Medicine* 13: 4310. https://doi.org/10.3390/jcm13154310.
12. Nahra, Raquel, Darvish, Shahrzad, Gandhi, Snehal, Gould, Suzanne, Floyd, Diane, Devine, Kathy, Fraimow, Henry, Dibato, John E., and Jean-Sebastien Rachoin. 2024. Impact of Povidone Application to Nares in Addition to Chlorhexidine Bath in Critically Ill Patients on Nosocomial Bacteremia and Central Line Blood Stream Infection. *Journal of Clinical Medicine* 13: 2647. https://doi.org/10.3390/jcm13092647.
13. Moppert, Justyna, Łężyk-Ciemniak, Eliza, and Małgorzata Pawłowska. 2024. Encephalitis in the Course of HHV-7 Infection in an Infant. *Journal of Clinical Medicine* 13: 418. https://doi.org/10.3390/jcm13020418.
14. Popa, Gabriela Loredana, Popa, Alexandru Cosmin, Mastalier, Bogdan, Crețu, Carmen Michaela, and Mircea Ioan Popa. 2023. Complicated Clinical Course of a Patient with Multivisceral Cystic Echinococcosis Requiring Extensive Surgical and Medical Treatment. *Journal of Clinical Medicine* 12: 5596. https://doi.org/10.3390/jcm12175596.
